# Accelerated and KWIC-filtered cardiac T_2_ mapping for improved precision: proof of principle

**DOI:** 10.1186/1532-429X-17-S1-W30

**Published:** 2015-02-03

**Authors:** Emeline Lugand, Jérôme Yerly, Hélène Feliciano, Jérôme Chaptinel, Matthias Stuber, Ruud B van Heeswijk

**Affiliations:** 1 Radiology, University Hospital (CHUV) and University (UNIL) of Lausanne, Lausanne, Switzerland; 2 Center for BioMedical Imaging (CIBM), Lausanne, Switzerland

## Background

**Figure 1:**
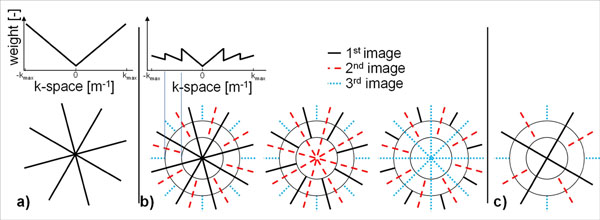
Schematic overview of radial k-spaces and their DCF. a) A normal radial k-space sampling pattern with above it the DCF along one radial line, which will be used to weigh the k-space points when regridding before the Fourier transform. Because of their high density, data from the center of k-space have a much lower weight than data from the outside. b) Three similar k-spaces that share their periphery through the KWIC filter, thus increasing the local sampling density and decreasing the local DCF (which in turn prevents noise amplification). The circles (determined with the Nyquist criterion) indicate the radii outside of which an extra k-space is added. c) An undersampled KWIC-filtered k-space. While the number of lines has decreased, the periphery of k-space still has a higher sampling density.

In recent years, several successful variations of T_2_-prepared cardiac T_2_ mapping techniques have been described for the quantification of cardiac edema [1,2,3]. Radial imaging for high-resolution T_2_ mapping [3] has the advantage of reduced motion sensitivity, but suffers from a lower signal-to-noise ratio (SNR) due to the undersampling of the periphery of k-space and the density compensation function (DCF) that increases the weight of the relatively noisy and less densely sampled k-space periphery (Fig.1a). Since the contrast of an image is defined by the center of k-space, a KWIC (k-space weighted image contrast [4]) filter can be used to share only the periphery among radial images that have the same geometry and different contrast, such as those used to generate a T_2_ map (Fig.1b). Furthermore, when undersampling is used to acquire more images per T_2_ map (resulting in more k-space peripheries that can be shared), KWIC filtering further reduces the noise-like undersampling artifacts (Fig.1c). The goal of this study was therefore to test whether the use of the KWIC filter leads to a higher precision in radial T_2_ maps for a given acquisition time.

## Methods

Standard navigator-gated radial T_2_ maps at 3T (Siemens Skyra) were acquired with 3 T_2_Prep durations, 308 radial lines with golden-angle increment per image, matrix 256x256, and spatial resolution (1.17mm)^2^[3] in an agar-NiCl_2_ phantom with relaxation times that approximated myocardium and blood. Subsequently, T_2_ maps at the same location were acquired with 6 T_2_Prep durations, with 308 and 110 lines per image. T_2_ maps were reconstructed both with and without the KWIC filter. The T_2_ standard deviations (σ_T2_) of the myocardial compartment in the resulting T_2_ maps were then compared. Finally, the same protocol was applied for the myocardium in 3 healthy volunteers.

## Results

**Figure 2:**
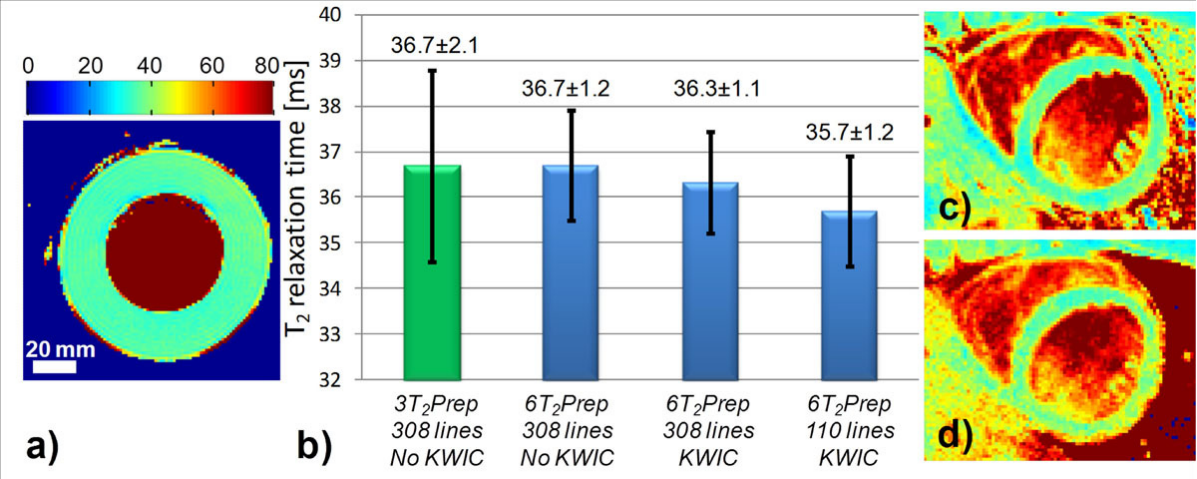
Radial T_2_ mapping with the KWIC filter. a) Example of a T_2_ map of the phantom, with a homogeneous T_2_ in blue-green circular compartment that has relaxation times similar to those of the myocardium. b) Results of the different T_2_ mapping techniques in the phantoms. The KWIC-filtered image that uses ~36% of the radial lines (and thus 72% of the acquisition time) has a significantly lower T_2_ standard deviation. c and d) Short-axis cardiac T_2_ maps in volunteers demonstrate equivalency of the undersampled images.

All phantom T_2_ maps could be successfully reconstructed (Fig.2a). The KWIC-filtered and undersampled T_2_ map was acquired in 72% of the standard acquisition time, and resulted in a σ_T2_ decrease from 2.1 to 1.2ms (Fig.2b). The acquisitions in the volunteers were similarly successfully reconstructed, and the resulting T_2_ values and σ_T2_ for the KWIC-filtered T_2_ maps (38.7±3.3ms) did not differ from the standard T_2_ maps (37.6±3.1ms, Fig. 2c&d).

## Conclusions

The phantom study demonstrated that the application of the KWIC filter to radial T_2_ mapping allows for a shortening of the acquisition time together with an increase in precision. The equivalency of the KWIC-filtered protocol *in vivo* might be caused by the lower overall SNR that is exacerbated by the undersampling, although this needs to be investigated in a larger cohort.

## Funding

Emma Muschamp Foundation (RBvH).
